# Superior oat (*Avena sativa*) genotypes enhance growth performance and nutrient efficiency in Bonga lambs fed total mixed rations

**DOI:** 10.1016/j.vas.2026.100879

**Published:** 2026-09-24

**Authors:** Kibreab Yosefe Wodebo, Taye Tolemariam Ejeta, Weyessa Garedew Terefe, Muluken Zeleke Ekule, Jane Ambuku Wamatu

**Affiliations:** a College of Agriculture and Veterinary Medicine, Jimma University, P.O.Box 307, Jimma, Ethiopia; b South West Ethiopia Agricultural Research Institute, Bonga Agricultural Research Center, South West Ethiopia Regional State, P.O.Box 101, Bonga, Ethiopia; c Ethiopian Agricultural Transformation Inistitute (ATI), P.O.Box 708, Addis Abeba, Ethiopia; d Wageningen University & Research, Animal Sciences Group, Wageningen, The Netherlands; e Internationa Center for Agricutera Research in dry Areas, P.O.Box 5689, Addis Abeba, Ethiopia

**Keywords:** Bonga lamb, Avena sativa, Nutrition, Growth, Nutrient intake, Digestibility, Feasibility

## Abstract

Evaluating forage genotypes is crucial for mitigating nutritional deficiencies and optimizing production in tropical small horn systems. This study investigated the effects of incorporating different Avena sativa genotypes (5526A, SRCPX80AB2806, and 5527A) into total mixed rations on nutrient intake, apparent digestibility, growth performance, and economic returns in yearling Bonga lambs under specific experimental conditions. Thirty intact male yearling lambs were assigned to three dietary treatments in a randomized complete block design over a 90-day feeding trial. The diets were formulated to be isocaloric and isonitrogenous (18.5% crude protein and 10.34 MJ/kg metabolizable energy).

The results demonstrated that genotype 5527A (Treatment 3) significantly improved dry matter intake, apparent digestibility, and average daily gains compared to the other evaluated genotypes. Specifically, lambs on the 5527A-based diet exhibited superior average daily gains and enhanced feed conversion efficiency. Furthermore, partial budget analysis indicated higher net economic returns under the conditions of this trial. However, these findings are limited to the specific breed, forage management practices, and production system evaluated. While these results highlight the potential of utilizing superior forage genotypes to improve smallholder productivity, further multi-environment evaluations are necessary before broader generalizations can be made.

## 1 Introduction

Livestock production plays a foundational role in the agricultural economy and food security of developing nations, particularly in sub-Saharan Africa, where smallholder mixed crop-livestock systems predominate (Ebro et al., 2024; Sileshi et al., 2001). In Ethiopia, the livestock sector contributes approximately 40% of the total national Gross Domestic Product (GDP) and supports the livelihoods of millions of smallholder farmers (Sileshi et al., 2001). However, national animal productivity remains severely constrained by structural nutritional limitations and profound, chronic feed shortages (Tegegne & Feye, 2020). Empirical evidence indicates that aggregate national feed resources fall short of satisfying the annual maintenance and production requirements of ruminants for dry matter, metabolizable energy, and crude protein by 21%, 52%, and 48%, respectively (Ashagrie et al., 2023). This systemic feed deficit which impacts between 79% and 99% of smallholder farming households annually is driven by progressive land use conversion, diminishing natural grazing pastures, and an over reliance on fibrous, low quality crop residues characterized by deficient nitrogen concentrations and low digestibility (Bezabih et al., 2016; Hussein et al., 2024; Melesse et al., 2023). Consequently, small ruminant growth rates and carcass yields in Ethiopia lag 25% to 30% behind regional East African averages, underscoring an urgent need for targeted nutritional interventions (Atsbha et al., 2021; Kochare et al., 2018).

To bridge these severe nutritional gaps and enhance the efficiency of tropical small ruminant production systems, optimizing forage resource utilization through the introduction of high yielding, nutritive cultivated forages is critical (Bezabih et al., 2016). Among cool-season annual cereal forages, oats (*Avena sativa L*.) have emerged as a premier dual-purpose crop capable of supplying high-quality biomass, digestible energy, and vital protein necessary to sustain optimal livestock performance during critical feed scarcity periods (Ahmad et al., 2014; Peterson et al., 2005; Ranjan et al., 2024). Beyond its favorable crude protein profile, *Avena sativa* is widely recognized for high palatability, rapid rumen fermentation kinetics (Kaya & Akçura, 2021; Prasanthi et al., 2025; Verma et al., 2023).

Despite the recognized general benefits of forage oats, a critical evaluation of recent literature reveals a major research gap: past agronomic and nutritional evaluations in East Africa have largely treated *Avena sativa* as a homogenous feed resource, failing to systematically connect specific genetic variations in structural cell wall architecture (such as neutral detergent fiber, acid detergent fiber, and lignin fractions) with subsequent animal intake dynamics, digestive efficiency, and meat production parameters (Gadisa et al., 2023; Mengistu et al., 2021; Tulu et al., 2020). Genotypic variations among oat cultivars directly dictate crucial physiological traits, including voluntary dry matter intake, rate of passage, total-tract nutrient digestibility, and daily weight gain in finishing sheep (Kafilzadeh & Heidary, 2013; Mut et al., 2015).

Recent multi-environment trials in Ethiopia have demonstrated significant phenotypic and nutritional variability among newly released and evauated *Avena sativa* genotypes (such as ILRI-5527A, SRCPX80AB2806, and ILRI-5526A), revealing substantial differences in dry matter yield, organic matter content, and cell-wall lignification when cultivated under highland tropical conditions. The selection of these three specific *Avena sativa* genotypes for this study is scientifically anchored in their distinct agronomic performance and divergent cell-wall structural profiles identified during recent regional adaptation trials in the South West Ethiopian highlands. Specifically, genotype ILRI-5527A exhibits superior agronomic adaptation coupled with lower structural fiber (NDF and ADL) and higher crude protein concentration compared to intermediate-performing lines like SRCPX80AB2806 and lower-performing genotypes such as ILRI-5526A (Wodebo et al., 2023). However, while their agronomic adaptability has been documented, the extent to which these specific genotypic disparities in structural fiber and nutrient fractions translate into associative feeding effects, voluntary intake, growth kinetics, and economic profitability when formulated into total mixed rations (TMR) for indigenous yearling sheep (such as Bonga lambs) remains largely unexplored.

Given that poor plane of nutrition leads to severe reproductive inefficiency, extended fattening periods, and economic losses for smallholders in zones like Kaffa, southwestern Ethiopia (Fesseha et al., 2023; Tilaye et al., 2022), identifying and deploying superior forage genotypes directly contributes to solving national feed scarcity at the farm gate. Therefore, this study was grounded on the hypothesis that selecting superior *Avena sativa* genotypes with optimized structural carbohydrate profiles (specifically genotype ILRI-5527A) would enhance voluntary feed intake, total-tract nutrient digestibility, and average daily gain in Bonga lambs without requiring additional external nutritional inputs. Consequently, the primary objective of this study was to evaluate the nutrient intake, apparent digestibility, growth performance, and economic profitability of yearling Bonga lambs fed total mixed rations incorporating three distinct *Avena sativa* genotypes (ILRI-5527A, SRCPX80AB2806, and ILRI-5526A) blended with a standard concentrate supplement.

## 2 Materials and methods

### 2.1 Description of the study area and ethics statement

The experiment was conducted at Bonga Agricultural Research Center, Modeyo Bonga Lamb multiplication substation located in Decha district, Kaffa Zone of South West Ethiopia. The center is found at 455 km from Addis Ababa at an altitude of 1809 m.a.s.l within latitude of N07°11′513’’ and a longitude of E036°18′052’’. The forages used in the feeding trials were planted and collected from the Alargeta substation of the research center. The study was conducted according to the guidelines of the declaration of directive 2010/63/EU of the European Parliament and of the Council of 22 September 2010 on the protection of animals used for scientific purposes. Following the above-mentioned guidelines, the study was checked and approved by Department of Animal Science of Jimma University, College of Agriculture and Veterinary Medicine, Research and the Ethical Review Committee (Jimma, Ethiopia; Ref.No: R: R/GS/922/2024).

### 2.2 Feed preparation and treatment allocation to experimental animals

Land was ploughed three times before the sowing at 20 cm spacing by drilling into the soil. Sowing was at a depth of 1.25 to 2.5 cm in moist soil to promote rapid germination at early rainy season of June. Sowing was at seed rate of 100 kg/ha using 100 kg/ha NPS and urea fertilizers were applied at two splits with a rate of 100 kg/ha. Weeding was done three times from sowing up to maturity stage. During milk stage harvested and keeps drying in shade and store as hay until feeding trial complete.

Three *Avena sativa* (ILRI_5527A, ILRI_5526A and AB2806) were harvested at the milk stage and wilted for hay preparation in open air under a shade. Hay that is adequate for the three months of feeding trial, one week of digestibility trial and two-week of acclimatization period was prepared. The hay was manually chopped into 1 cm forming hay before mixing with concentrate. 1 cm choping length was intentionally selected to reduce the physical ability of the animals to sort. Dietary components were formulated to ensure that despite the 1 cm chop length and high concentrate level, the estimated physically effective NDF (peNDF) concentration met the minimum requirements necessary to maintain adequate rumination and stable rumen pH in lamb (minimum 15–19% DM basis; NRC, 2007). Particle efective NDF(peNDF) values were 29.39%, 29.51%, and 28.76% (DM basis) for T1, T2, and T3, respectively. Ultimately, all three treatments successfully meet the critical 15–19% DM minimum threshold required to maintain optimal rumen health, maintain stable ruminal pH, and support efficient lamb performance despite the challenges of high-concentrate formulations.This was achieved by selecting forage components with high structural integrity and managing a gradual transitional adaptation phase to stabilize the ruminal environment.

The experimental feeding trial was structured around three distinct dietary treatments (T1, T2, and T3) designed to evaluate the effects of different *Avena sativa* varieties under isocaloric and isonitrogenous on a dry matter basis with fixed ingredient ratios (T1=25% *Avena sativa* 5526A and 75% concentrate, T2=30% *Avena sativa* SRCPX80AB2806 and 70% concentrate, T3=30% *Avena sativa* 5527A and 70% concentrate) on lamb performance. The above percentage make the daily amount of feed 196.01 *g* Avena sativa 5526A+645.88 g Concentrate mix (T1), 235.21 *g* of Avena sativa SRCPX80AB2806+ 602.82 *g* of concentrate mix (T2) and 235.21 g of Avena sativa 5527A+ 602.82 g of concentrate mix (T3).To ensure adequate mineral supplementation across all cohorts, every treatment was uniformly fortified with 1% salt and 1% limestone. 5% concentrate adition on T1 over the rest two treatments(T2&T3) and decreasing 5% avena sativa 5526A from daily feeding compared to the rest treatments(T2&T3) make all the three treatments isocaloric and isonitrogenous.

Crucially, all three dietary regimens were formulated to be both isocaloric and isonitrogenous based on the analyzed values of the individual ingredients, maintaining a consistent metabolizable energy (ME) content of 10.34 MJ/kg and a crude protein (CP) level of 18.5%, thereby isolating the specific variety of *Avena sativa* as the primary experimental variable. The commercial concentrate used comprised of 69% barley bran, 29% oil seed cake developed to fulfill the essential nutrient requirement of Bonga Lamb . To ensure ad libitum intake, the ingredients were thoroughly mixed as a Total Mixed Ration (TMR) and offered daily ensuring a target of approximately 10% refusals. Refusals from the previous 24-hour period were collected, weighed, and recorded daily at 07:30 h prior to feeding to determine daily dry matter intake (DMI). The amount of feed offered to each individual animal was adjusted daily at the morning feeding, calculated based on the previous day’s actual intake. The mineral balances (Ca and P) of the TMRs were verified analytically using AOAC (2005) method. The analyzed dietary Ca:P ratio across the treatments was 2:1.

The treatment diets are designed to fulfill the optimum recommended required CP and energy of the experimental lamb (112 g CP/kg (62 g digestible protein) and 10.34 MJ ME/kg DM (Kearl, Kearl, L.C., 1982., 1982). The experiment was conducted following the specific recommendations of the NRC (2007) for the nutritional requirements of growing sheep, specifically utilizing nutrient requirement models for a 30 kg growing ram lamb targeted at an average daily gain (ADG) of 120 g/day. This included meeting or exceeding recommended allowances for metabolizable energy (10.34 MJ/kg DM), crude protein (18.5% DM), physically effective neutral detergent fiber (peNDF minimum threshold of 15–19% DM to maintain stable ruminal pH), and mineral balances (specifically 5.9 g calcium and 3.2 g phosphorus daily. Total mixed rations were offered in two equal meals daily (at 08:00 h and 16:00 h) to prevent binge-feeding and stabilize ruminal pH. The entire quantity of concentrate mixture required for the duration of the entire trial was procured and thoroughly blended in one single, uniform batch at the initiation of the study. The concentrate batch was stored in a dry, well-ventilated facility under uniform environmental conditions.

### 2.3 Experimental design and management of the experimental animals

A total of 30 heathy yearling intact male (ram) lambs Bonga lamb breed (30.98 ± 1.18 kg) obtained from Bonga lamb Multiplication substation station with recorded history of age and weight were used to conduct this experiment. These were blocked in to 10 and three treatments assigned. Each groups was kept for one month of quarantine and 15 days of acclimatization period of their specific assigned diets to manage acidosis alowing adaptation to high-concentrate diets to the experimental feed as well as new facilities. 90 days of feeding trials evauation was done and sequencialy 7 days of digestibility evaluation after feeding trial competed (Abera et al., 2018). The experiment done following the procedure of (NRC, 2007) and based on data from a chemical analysis of feed performed previously during the acclimation period. The lambs were weighed every ten days, on the same date and time before morning feeding using digital balance. To treat internal and external parasites, the lams were given Albendazole bolus acarimic spray. The lambs were also given vaccinations against pneumonia, lamb pox, blackleg and anthrax.

During the feeding trial, animals were fed their individual diets according to the experimental scheme in separate feeding troughs. The animals had free access to clean drinking water throughout the experimental period. The experiment was laid out in a randomized complete block design (RCBD) with three treatments replicated ten times. Within each block, animals were randomly assigned to one of the three dietary treatments using a random number generator in R. Each animal was housed in well ventilated individual pen equipped with feeding and watering troughs. They were randomly housed in individual holding pens (1.5 × 2.5 m^2^) with concrete floors on an open-air platform (Holmøy et al., 2012).

### 2.4 Growth performance and feed consumption

Feeds offered and refusals were recorded every day during the experimental period. Feed intake was determined as the difference between offer and refusal. The initial and final weights were taken two times on consecutive days, and the averages of the two were taken as the initial and final weights, respectively (Nurfeta, 2010). Average daily gain (g) was calculated by dividing the difference between final and initial weights by the number of days (90).

### 2.5 Chemical analysis of feed and feces

All chemical analyses were carried out in triplicate. The feces and feed samples were analyzed for dry matter (DM), ash, and ether extract (EE) according to AOAC (2005). Ash was determined by combusting the sample at 550 °C for 5 h. Neutral detergent fiber (NDF), acid detergent fiber (ADF), and Acid detergent lignin (ADL) were determined according to Van Soest et al. (1991).

The IVOMD of the forage samples was determined using the two-stage in vitro rumen liquor-pepsin procedure described by Tilley and Terry (1963). Rumen liquor (inoculum) was collected from ruminally fistulated lamb fed a standard maintenance diet. The fluid was strained through four layers of surgical gauze into a pre-warmed thermos flask under anaerobic conditions (CO_2_). For the first stage, samples were incubated with a mixture of rumen liquor and McDougall’s artificial saliva buffer for 48 h at 39 °C under continuous agitation. In the second stage, the residue was incubated with a pepsin-hydrochloric acid (HCl) solution for a further 48 h to simulate abomasal digestion. Residual organic matter was measured by ashing. The in vitro dry matter digestibility (IVDMD) and organic matter digestibility (IVOMD) of the ingredients and total mixed rations (TMR) of treatments samples were determined using the two stage rumen fluid pepsin digestion technique described by Tilley and Terry (1963). The digestible organic matter content in the dry matter (DOMD, g/kg DM; or d-value) obtained from this analysis was subsequently used to estimate the Metabolizable Energy (ME, MJ/kg DM) content of the diets using the standard MAFF (1993) and AFRC (1993) predictive equations (ME = 0.016 * DOMD). IVOMD (and subsequent ME calculation) was performed independently on both the individual hay samples and the final TMR treatments. Nitrogen (N) was analyzed according to the Kjeldhal procedure, and CP was determined as N × 6.25 (Ma *et al*., 2015).

### 2.6 Digestibility trial

Following the 90-day feeding trial, animals from each treatment group were fitted with harnessed fecal collection bags for a 7 day collection period (preceded by a 3 day adaptation to the harnesses/cages). During the collection period, fecal bags were checked four times daily (at 06:00, 12:00, 18:00, and 24:00 h) to ensure total fecal recovery and prevent sample degradation. Total feces were collected daily, weighed, and sampled to determine nutrient digestibility. The daily feces excreted by each animal were weighed and 20% was subsampled, pooled on the individua lamb basis, and frozen (−20 °C) pending to chemical analysis (Bosshard *et al*., 2011). For statistical modeling, nutrient intake and fecal excretion values were averaged over the 7-d collection window per individual animal to compute a single apparent total-tract digestibility value per experimental unit. Feed leftovers were removed daily, each morning at 8:00 AM and weighed. During the collection period, samples of feed and refusals were collected, composited with the animal, ground to pass through 1 mm screen for laboratory analysis (Romero *et al*., 2000). For chemical analysis feed and feces samples were dried at 60 °C for 72 h to constant weight, pulverized to pass through 1 mm sieve screen and stored pending to chemical analysis and kept in screw-capped plastic bottles until analysis (ARC, 1980). At the start and end of the digestibility trial, each animal was individually weighed before the morning feeding. At the end of the experiment, all the experimental lambs were fasted for 12 h, and weighed (Fitwi & Tadesse, 2013).

During the digestibility data collection period, daily feed offered, refusal, and feces voided were weighed and recorded. Samples of feed offered, feed refusal and feces of each animal were taken each day in the morning, and weekly composite samples were formed. To maintain sample integrity, any collection intervals showing evidence of urine contamination or feed spillage into the bag were discarded from the daily total, and the animal’s daily excretion was calculated based on the remaining uncontaminated intervals. Following standard Penn State Particle Separator (PSPS) protocols (peNDF = NDF *pef), the total mixed rations (TMR) were sieved through a 10 mm screen. A pef value of 0.70 was assigned based on the 10 mm theoretical length of cut (TLOC) of the mixed diets.

The apparent digestibility (AD) of DM, OM, CP, NDF, and ADL were calculated using the following equations suggested by McDonald et al. (2010).

(1) $$\text{Apparent}\;\text{DMD}=\frac{\left(DM\;intake-Fecal\;DM\;output\right)}{DM\;intake}*100$$

(2) $$Apparent\;nutrient\;DC=\;\frac{\left(Nutrient\;intake-Fecal\;Nutrient\;output\right)}{Nutrient\;intake}*100$$

Dry Matter, Ash, and Ether Extract (EE): Analyzed following standard procedures outlined by AOAC (2005). Neutral Detergent Fiber (NDF), Acid Detergent Fiber (ADF), and Acid Detergent Lignin (ADL): Determined sequentially using the detergent fiber system described by Van Soest et al. (1991). In Vitro Organic Matter Digestibility (IVOMD) & d-Value: Determined using the two-stage rumen fluid-pepsin technique established by Tilley and Terry (1963), with Metabolizable Energy (ME) estimated via standard predictive equations (MAFF, 1993; AFRC, 1993). Apparent Total-Tract Digestibility: Calculated using total fecal collection methods and standard nutrient balance equations outlined by McDonald et al. (2010).

### 2.7 Partial budget analysis

Partial budget analysis was performed by considering variable costs i.e. experimental animal market price and feed, labor, veterinary, and total revenue (income) from sales of animals. Net income obtained from the experiment was calculated as the difference between total revenue (total returns) and total variable costs according to the formula shown below and developed by (Upton, 1979). A partial budget analysis was executed to evaluate the economic feasibility of utilizing the different forage genotypes, following the standard methodology of CIMMYT (1988). To maximize real-world generalizability, the economic analysis explicitly accounted for the agronomic production costs and dry matter (DM) yields per hectare for each specific forage genotype.

The total variable costs (TVC) included both agronomic cultivation inputs (seed cost, fertilizer, land preparation, harvesting labor, and cost per kg DM) and animal management inputs (concentrate costs, veterinary care, and labor). Cost per kg gain from the formua of total feed cost/ total weight gain in kg (Ates et, a., 2015). Farm-gate prices used to value the forage and the inputs. Because the harvested Avena sativa biomass was utilized directly within the feeding trial, internal transfer costs were computed directly from local dry-matter market valuations (0.36USD/kg), avoiding any omission of forage production expenses (Tilaye et al., 2022; Ebro et al., 2024).

(3) NI=TR−−TVC

(4) ΔNI=ΔGR−−ΔTVC

(5) MRR=ΔNI/ΔTVC

Where NI = net income, TR = Total return, TVC = Total variable cost, ΔNI = change in net income, ΔGR = change in gross return, ΔTVC = change in total variable cost, MRR = marginal rate of return. Marginal rate of return (MRR) is another way of considering the cost factor. It measures the ∆NI which is generated by each additional unit of expenditure (∆VC).

The price of the experimental animals was estimated using the market price during purchase and selling price at the end as the source of income, without including income from manure in the total revenue. All the calculations were made in US dollar through conversion of the Ethiopian Birr (ETB) using an exchange rate at a time of conducting the experiment. Live animal pricing at each period was carried out using information from five local livestock merchants. Return from *Avena sativa* production was not included in a cost becouse produced *Avena sativa* used directly for feeding by the experiment for evaluation.

### 2.8 Statistical analysis

Data collected for intake, digestibility, and growth performance were evaluated using Linear Mixed Models (LMM) in R (v.4.4.2), treating dietary genotype as a fixed effect and experimental block (stratified by initial body weight) as a random effect. Prior to parametric analysis, model residuals were formally tested for normal distribution using the Shapiro-Wilk test, and homoskedasticity was verified via Levene’s test. Post-hoc comparisons of treatment means were conducted using Tukey's Honestly Significant Difference (HSD) test, with statistical significance declared at P < 0.05 (Kafilzadeh & Heidary, 2013; Mut et al., 2015)

The variations were modeled as follows:

(6) Yij=μ+Ti+Bj+Eij

Where; Y_ij_ is the response of the animal in blocks I given treatment j, μ is the overall mean, T_i_ is the fixed effect of the i^th^ dietary treatment, β_j_ is the random effect of the j^th^ block (based on initial body weight stratification), assumed to be independently and identically distributed as Bj ∼ N (0,$\;\sigma_{B}^{2}$), and εij is the random error term. Prior to statistical analysis, formal tests for model assumptions were conducted. Normality of the residuals was assessed using the Shapiro-Wilk test, while homoskedasticity was evaluated using Levene’s test. No severe violations of assumptions were detected.

## 3 Results

### 3.1 Nutritional profile and chemical architecture of diet substrates

The chemical compositions and ingredient profiles of the individual feed items and experimental total mixed rations (TMR 1, TMR 2, and TMR 3) formulated using different *Avena sativa* varieties combined with a concentrate mix are presented in Table 1. The experimental diets demonstrated a high degree of nutritional uniformity across treatments. Specifically, dry matter (DM) content among the TMR diets ranged from 88.21% to 88.62%, while organic matter (OM) and crude protein (CP) contents remained consistent, averaging 93.94% and 18.5%, respectively. Fiber fractions were well-balanced, with neutral detergent fiber (NDF), acid detergent fiber (ADF), and acid detergent lignin (ADL) spanning 38.34% to 39.34%, 19.91% to 20.50%, and 9.10% to 9.89%, respectively. Furthermore, all experimental diets delivered uniform metabolizable energy (ME) levels of 10.34 MJ/kg alongside consistent in vitro organic matter digestibility (IVOMD) ranging from 64.57% to 64.66%, indicating that substituting the different *Avena sativa* cultivars in these proportions successfully yielded isoenergetic and isonitrogenous rations suitable for comparative evaluation.

**Table 1:** Ingredient and chemical composition of the experimental diets.

SN	Feed Items	DM	EE	Ash	OM	CP	NDF	ADF	ADL	IVOMD%	ME	CA	P
*1*	*Avena sativa* 5526A	95.03	1.22	7.22	92.78	9.1	51.21	34.69	6.1	51.9	8.3	0.38	0.22
*2*	*Avena sativa* SRCPX80AB2806	95.03	1.18	6.01	93.99	11.05	49.07	32.73	5.23	52.8	8.44	0.41	0.25
*3*	*Avena sativa* 5527A	95.07	2.06	4.07	95.93	12.04	45.73	30.76	4.31	52.9	8.47	0.45	0.28
4	Nug seed cake=N	92.5	6.5	10.2	89.8	34.5	42.1	28.4	11.2	64.01	10.2	0.85	1.1
5	Barley bran=B	89.2	4.1	5.3	94.7	14.5	49.5	16.2	3.5	74.13	11.8	0.1	0.9
6	Concentrate mix (69:29, B:N)	97.15	3.01	5.45	96.76	21.27	35.18	15.27	11.16	71.82	11.49	0.32	0.91
7	TMR 1 (T1)	88.62	2.5	6.27	93.73	18.5	39.18	20.12	9.89	64.66	10.34	0.75	0.33
8	TMR 2 (T2)	88.21	2.64	6.1	93.9	18.5	39.34	20.50	9.38	64.57	10.34	0.73	0.34
9	TMR 3 (T3)	88.21	2.64	5.8	94.2	18.5	38.34	19.91	9.10	64.59	10.34	0.74	0.36

DM: dry matter; EE: Crude fat; OM: organic matter; CP: crude protein; NDF: neutral detergent fiber; ADF: acid detergent fiber; ADL: Acid detergent lignin; ME: metabolizable energy; TMR:Total mixed ration; T1=25% Avena sativa 5526A and 75% concentrate+1% sat and 1% limestone, T2=30% Avena sativa SRCPX80AB2806 and 70% concentrate+1% sat and 1% limestone, T3=30% Avena sativa 5527A and 70% concentrate+1% sat and 1% limestone.

### 3.2 Voluntary feed intake and nutrient consumption dynamics

The voluntary feed and nutrient intake dynamics of Bonga lambs fed on different *Avena sativa* genotypes are summarized in Table 2. Total dry matter intake (TDMI) was significantly higher (p < 0.05) in lambs fed the *Avena sativa* 5527A genotype mixed with concentrate (T3) compared to those fed diets based on genotypes 5526A (T1) and SRCPX80AB2806 (T2). Consequently, lambs in the T3 group demonstrated a significantly higher (p < 0.05) consumption of organic matter, crude protein (CP), crude fat (EE), and metabolizable energy (ME).

**Table 2:** Nutrients (g/d DM) and energy intake (MJ/kg DM) of Bonga lamb feed different *Avena sativa* hay with concentrate mix.

**Nutreints**	**T1**	**T2**	**T3**	**SE**	**p-value**
TDMI (g/d)	862.02^c^	940.99^b^	997.98^a^	0.01	2.2e-16***
DMI(g/kg BW^0.75^)	57.46^c^	61.49^b^	61.60^a^	0.017	< 2.2e-16
OMI(g/d)	809.05^c^	883.99^b^	936.98^a^	0.02	2.2e-16***
EEI(g/d)	16.00^c^	20.02^b^	29.03^a^	0.01	2.2e-16***
CPI(g/d)	158.96^c^	173.14^b^	186.42^a^	0.02	2.2e-16***
CPIRMBW(g/kg BW^0.75^)	10.60^a^	11.31^b^	11.51^a^	0.003	2.2e-16***
NDFI(g/d)	384.01^c^	404.99^b^	420.99^a^	0.01	2.2e-16***
ADFI(g/d)	198.98^c^	209.03^b^	211.99^a^	0.02	2.2e-16***
ADLI(g/d)	88.00^b^	85.97^c^	88.97^a^	0.02	2.2e-16***
MEI (MJ/kg DM)	9.07^c^	9.72^b^	10.31^a^	0.0001	2.2e-16***
MEIRMBW(g/kg BW^0.75^)	0.61^c^	0.63^b^	0.64^a^	0.0013	2.2e-16***

a,b,c Mean values with different superscripts in a row are significantly different at * = (P < 0.05), ** = (p < 0.01), *** = (p < 0.001). TDMI: total dry matter intake; OMI: organic matter crude protein intake; EEI: ether extract crude protein intake; CPI: crude protein intake; CPIRMBW: crude protein intake relative to metabolisable body weight; NDFI: neutral detergent fiber crude protein intake; ADFI: acid detergent fiber crude protein intake; ADLI: acid detergent lignin crude protein intake; MEI: metabolizable energyintake; MEIRMBW : metabolizable energy intake relative to metabolisable body weight; SE: standard error; SL: significance level; ns: nonsignificant.

Conversely, despite higher total dry matter consumption, the intake of structural fiber fractions specifically neutral detergent fiber (NDF) and acid detergent fiber (ADF) remained structurally proportionate, reflecting the varied chemical compositions of the respective oat genotypes. The lowest overall nutrient and energy intake was observed in the T1 group, indicating that the 5526A genotype had the most limiting effect on voluntary consumption.

### 3.3 Apparent digestibility of structural and soluble fractions

The results of apparent DM, OM, CP, NDF, and ADF digestibility are presented in Table 3. The apparent digestibility coefficients for dry matter (DM), organic matter (OM), crude protein (CP), ether extract (EE), and structural carbohydrate fractions (NDF, ADF, and ADL) exhibited highly significant variations (p < 0.001) among the experimental diets (Table 3). Lambs fed the T3 diet (comprising Avena sativa genotype 5527A combined with concentrate) recorded the highest apparent digestibility values across all measured parameters, with DM, OM, CP, NDF, and ADF digestibilities reaching 75.99%, 77.97%, 83.99%, 61.99%, and 54.01%, respectively. Conversely, the lowest apparent digestibility coefficients were consistently observed in the T1 group (*Avena sativa* genotype 5526A), where DM, OM, CP, NDF, and ADF digestibilities were restricted to 67.96%, 69.97%, 76.00%, 52.02%, and 44.01%. Intermediate digestibility values were recorded for the T2 dietary treatment. Overall, the mean apparent nutrient and fiber digestibility coefficients followed a parallel trend to the voluntary feed consumption data, demonstrating that superior intake in the T3 group was matched by enhanced ruminal degradation and total tract nutrient utilization.

**Table 3:** Apparent digestibility of nutrients of Bonga lamb fed different *Avena sativa* hay with concentrate mix (DM %).

**Digestibility (%)**	**T1**	**T2**	**T3**	**SE**	***p-*value**
DM	67.96^a^	71.04^b^	75.99^a^	0.02	< 2.2e-16***
OM	69.97^c^	74.02^b^	77.97^a^	0.02	< 2.2e-16***
EE	72.01^c^	76.99^b^	81.99^a^	0.02	< 2.2e-16***
CP	76.00^c^	80.00^b^	83.99^a^	0.02	< 2.2e-16***
NDF	52.02^a^	56.99^b^	61.99^a^	0.01	< 2.2e-16***
ADF	44.01^c^	49.00^b^	54.01^a^	0.01	< 2.2e-16***
ADL	0.99^a^	2.99^b^	4.99^a^	0.02	< 2.2e-16***

a, b, c Mean values with different superscripts in a row are significantly different at * = (P < 0.05), ** = (p < 0.01), *** = (p < 0.001).DM: dry matter; OM: organic matter; EE: ether extract; CP: crude protein; NDF: neutral detergent fiber; ADF: acid detergent fiber; ADL: acid detergent lignin; SE: standard error; SL: significance level; ns: nonsignificant.

### 3.4 In vivo growth kinetics

The growth performance and slaughter parameters of Bonga lambs subjected to the three dietary treatments are summarized in Table 4. Initial body weights (IBW) among the experimental groups were homogeneous (P > 0.05), averaging approximately 30 kg, which confirms the uniform background and baseline characteristics of the experimental animals. However, significant variations (P < 0.05) were observed in final slaughter body weight (SBW), metabolic body weight (MBW), total body weight gain (BWG), and average daily gain (ADG) across the treatment groups. Lambs fed the T3 diet (*Avena sativa* 5527A variety supplemented with concentrate) achieved the highest SBW (41.02 kg), BWG (10.36 kg), and ADG (114.95 *g*/d), followed in descending order by those on T2 (87.99 g/d) and T1 (70.00 g/d). The feed conversion ratio (FCR) improved significantly (P < 0.001) from 12.31 in T1 to 10.71 in T2 and 8.69 in T3. Furthermore, nutrient utilization efficiency metrics such as crude protein intake per kilogram of gain and metabolizable energy intake per kilogram of gain were significantly lowest in T3 (1621.75 g and 89.49 MJ, respectively), reflecting superior biological conversion efficiency of dietary inputs into body mass compared to T1 and T2.

**Table 4:** Slaughter performance of Bonga Lamb fed different *Avena sativa* varieties hay with concentrate mix.

Variables	T1	T2	T3	SE	*p-*value
IBW(kg)	29.96	30.04	30.07	0.05	0.3667
SBW(kg)	37.02^a^	38.01^b^	41.02^a^	0.02	< 2.2e-16***
MBW	15.0^c^	15.3^b^	16.2^a^	0.004	< 2.2e-16***
BWG (kg)	7.27^c^	8.92^b^	10.36^a^	0.02	< 2.2e-16***
ADG(g/d)	70.00^c^	87.99^b^	114.95^a^	0.02	< 2.2e-16***
FBCS	2.57^a^	3.70^b^	4.46^a^	0.02	2.2e-16***
FCR(g/g)	12.31^a^	10.71^b^	8.69^c^	0.009	< 2.2e-16 ***
GTFR(g/g)	4.66^c^	5.75^b^	7.09^a^	0.017	< 2.2e-16***
CP Intake per kg gain(g)	2270.87^a^	1967.73^b^	1621.75^c^	0.01	< 2.2e-16 ***
ME Intake per kg gain(MJ)	111.68^a^	103.94^b^	89.49^c^	0.05	< 2.2e-16 ***

a,b,c Mean values with different superscripts in a row are significantly different at * = P < 0.05, ** = p < 0.01, *** = p < 0.001. IBW: initial body weight; SBW: slaughter body weight; MBW: Metabolic body weight; BWG: Body weight gain, ADG: Average daily gain; FCR:Feed conversion ratio(DMI/ADG); GTFR:Gain to Feed Ratio =ADG/ Average Daily Feed Intake (ADFI); CP:Crude protein; ME:Metabolisable energy;DMI: Dry Matter Intake.

### 3.5 Correlation matrix and linear relationships among dietary intakes and performance variables

The multiple regression analysis at Fig. 1 demonstrates a profound physiological link between nutrient intake, diet utilization efficiency, and growth performance in the experimental lamb.

**Fig. 1:**
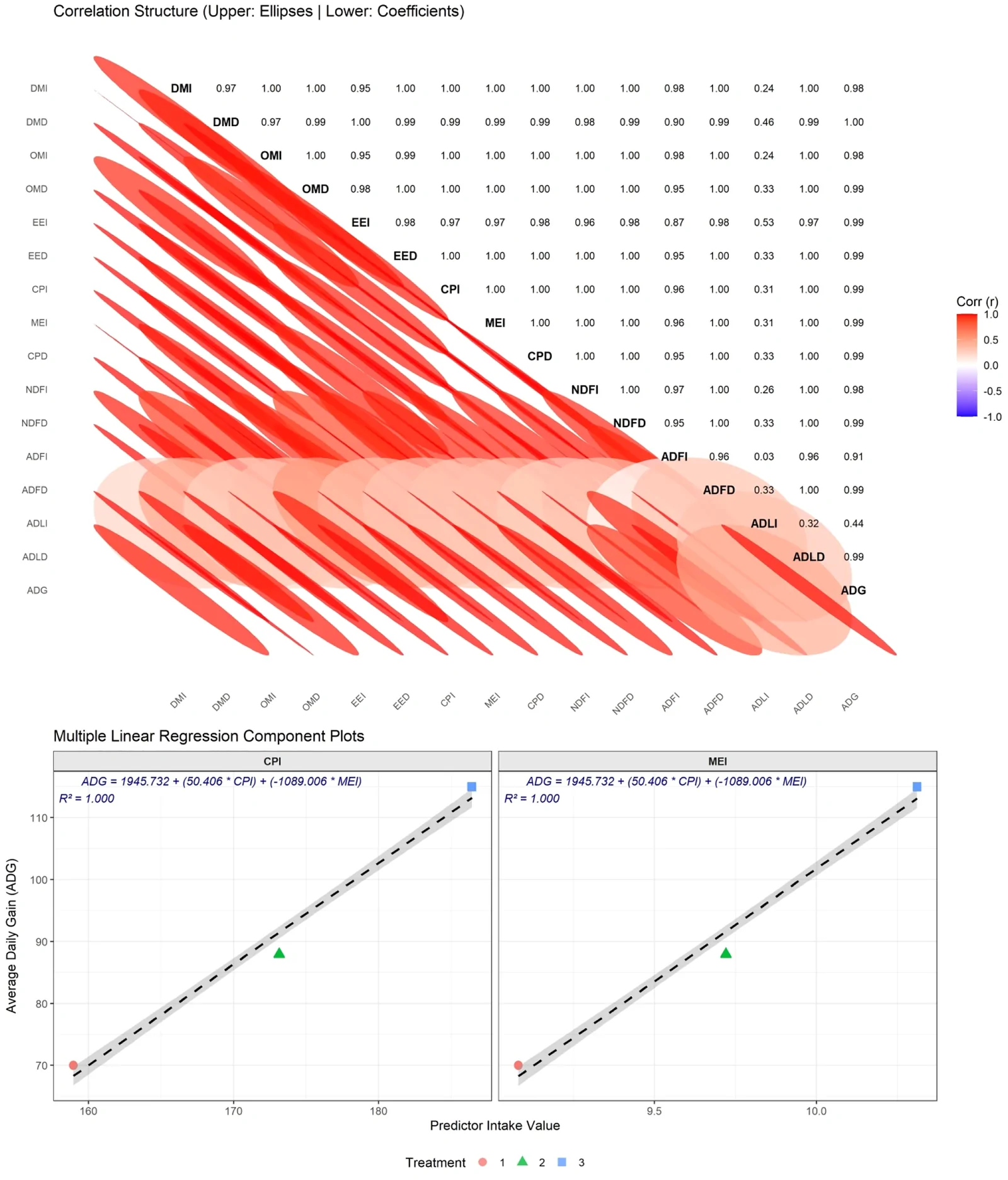
Multipanal plot of correation and regresion analysis. The regression lines and R2 values were derived using aggregated treatment level mean values (n = 3 dietary treatments: T1, T2, and T3).

The correlation matrix and multiple linear regression models illustrate the strong physiological coupling between nutrient intake parameters and growth performance in Bonga lambs. Correlation analyses reveal near-perfect positive linear associations (r ≥ 0.95) among total dry matter intake (TDMI), organic matter intake (OMI), crude protein intake (CPI), metabolizable energy intake (MEI), and average daily gain (ADG). The multiple linear regression component plots confirm that Average Daily Gain (ADG) is driven by Crude Protein Intake (CPI) and Metabolizable Energy Intake (MEI), yielding a deterministic fit (R^2^ = 1.000): ADG = 1945.732 + (50.406 *CPI) + (-1089.006*MEI). As dietary treatments transition from T1 to T3, the incremental elevation in nutrient consumption and total tract digestibility ensures that energy and amino acids are efficiently partitioned toward body weight gain rather than metabolic waste.

### 3.6 Bio-Economic viability and financial sensitivity scenarios

The results of partial budget analysis evaluation are presented in Table 5. The selling price of the lamb was estimated by six independent and experienced traders. Manure that may be used as fertilizer was not considered as a source of income during the calculations due to the fact that lamb manure is not commonly sold in the study area. The dry matter (DM) yield per hectare varied among the evaluated Avena sativa genotypes, with Avena sativa 5527A producing the highest yield at 12 t/ha, followed closely by Avena sativa SRCPX80AB2806 at 11.67 t/ha, and Avena sativa 5526A at 9.5 t/ha. Economic valuation of this biomass is priced at 15 ETB (approximately 0.36 USD) per kilogram of dry matter.

**Table 5:** Partial budget analysis of bonga lamb fed different *Avena sativa* hay mixed with concentrate.

Variables(USD)	T1	T2	T3
Number of Animals	10	10	10
Gross income (finished lamb price) (A)	83.89	101.85	123.04
Purchase price of animals (C)	44.55	44.55	44.55
Cost of labour:land preparation, sowing, weeding, and harvesting(D)	32.28	45.20	38.08
Cost of fertiizer(E)	3.38	4.73	3.42
Cost of *Avena sativa* consumed (F)	0.07	0.08	0.08
Cost of concentrate (Lamb /90 days) (G)	0.86	0.82	0.82
Cost of salt(H)	0.01	0.01	0.01
Cost of limestone(I)	0.001	0.001	0.001
Costs of labor: chopping & animas management (J)	2.32	2.32	2.32
Costs of the vaccine (K)	0.01	0.01	0.01
Total variable Cost(TVC)=B(C+D+E+F+G+H+I+J+K)	83.48	97.73	89.29
Net benefit (A-B)	0.41	4.13	33.75
Marginal rate of return (MRR)=A/B	1.01	1.04	1.38
Feed Cost per kg Gain (Total feed cost/Live weight gain in kg)= Cost per kg Gain = FCR * Cost of Feed per kg	0.13	0.10	0.08

TVC: Total Variable cost; MRR: Marginal rate of return; USD: United States of America dollar; 1USD=41.47 Ethiopian birr at the start of the experiment and 41.47 Ethiopian birr at the last day of experiment. SE: standard error; SL: significance level; ns: nonsignificant.

The partial budget analysis evaluated the economic feasibility of feeding Bonga lambs total mixed rations formulated with different Avena sativa varieties and a concentrate mix (Table 5). Total variable costs (TVC) varied across treatments due to differences in agronomic labor, fertilizer requirements, and biomass purchase costs, amounting to 83.48 USD for T1, 97.73 USD for T2, and 89.29 USD for T3. Gross income, determined by the finished market price assigned by experienced livestock traders, increased progressively from 83.89 USD in T1 and 101.85 USD in T2 to a high of 123.04 USD in T3. Consequently, net economic benefits increased substantially across the dietary treatments. Treatment 3 yielded the highest net benefit of 33.75 USD per animal, compared to 4.13 USD for T2 and a marginal 0.41 USD for T1. The marginal rate of return (MRR) followed a similar trend, registering 1.38 for T3, whereas T1 and T2 recorded lower returns of 1.01 and 1.04, respectively. Furthermore, feed cost per kilogram of gain decreased favorably from 0.13 USD in T1 and 0.10 USD in T2 down to 0.08 USD in T3.

The sensitivity analysis (Fig. 2) assessed the financial resilience of each dietary treatment under simulated adverse market fluctuations, including incremental increases in total variable costs (10% and 20%) and decreases in gross income (10% and 20%), as well as combined shock scenarios. The evaluation demonstrates that Treatment 3 maintains a strongly positive net financial benefit across all tested economic stress scenarios, outperforming T1 and T2 which frequently fall into net economic losses under market contraction.

**Fig. 2:**
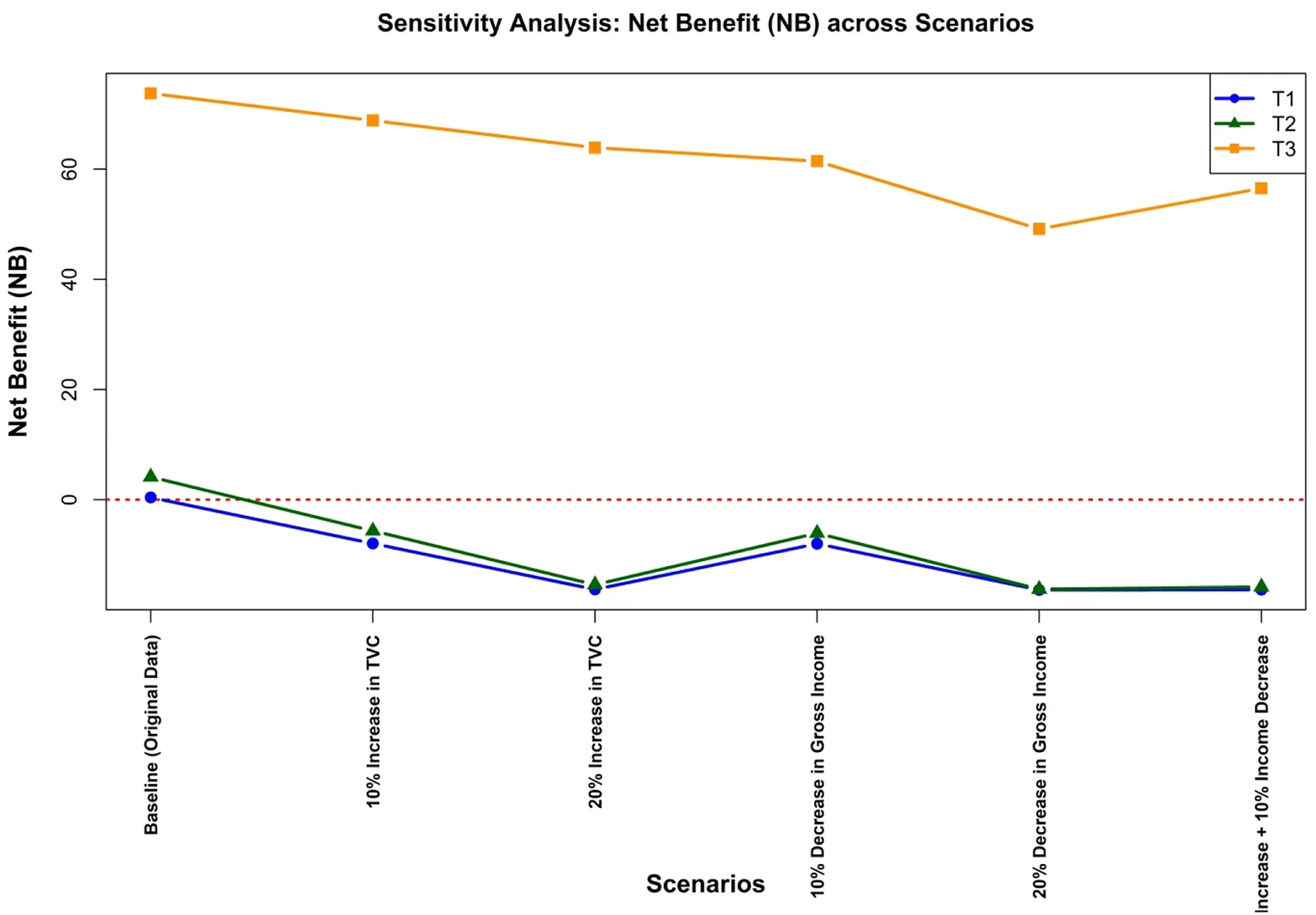
Sensiitivity anaysis.

## 4 Discussion

### 4.1 Nutritional profile and chemical architecture of diet substrates

Composite samples of the final TMRs collected weekly throughout the trial demonstrated that the treatment diets successfully met the nutritional requirements of yearling Bonga lambs in terms of crude protein (CP) and metabolizable energy (ME). The observed variations in the chemical composition of the *Avena sativa* genotypes with CP ranging from 9.10% to 12.04% DM and ME ranging from 8.30 to 8.47 MJ/kg DM align with or expand upon ranges documented in previous studies across different production environments. For instance, the CP values observed in this study exceed the ranges reported by Kebede et al. (2020); Tulu et al. (2020), Gadisa et al. (2023), and Mengistu et al. (2021), while remaining comparable to findings by Wayou et al. (2019) and lower than the upper thresholds noted by Khan et al. (2014). Similarly, the organic matter (OM) percentage of the oat genotypes ranged from 92.78% to 95.93%, matching or exceeding values reported in prior regional and international evaluations (Saman, 2003; Wada et al., 2019; Kuter et al., 2023; Ma et al., 2021; Kaba et al., 2020). From a biological perspective, the minor yet critical differences in cell-wall architecture among the tested oat genotypes dictate their nutritional quality and subsequent utilization by ruminants. The neutral detergent fiber (NDF) content of the oat varieties ranged from 45.73% to 51.21%, which falls favorably below higher values reported for alternative cultivars (Kafilzadeh et al., 2013; Ma et al., 2021), though certain varieties exhibited comparable structural profiles to established standards (Mut et al., 2015). Acid detergent fiber (ADF) ranged from 30.76% to 34.69%, aligning closely with or improving upon broader literature ranges (Ahmad et al., 2014; Keba et al., 2020). Furthermore, acid detergent lignin (ADL) concentrations ranged from 4.31% to 6.10%, reflecting variations that directly influence structural recalcitrance. Explaining the biological relevance of these structural differences, genotypes possessing lower NDF and fiber contents (such as Avena sativa ILRI_5527A in T3) present a less restrictive matrix for digestion. Lignin acts as a physical barrier encrusting cellulose and hemicellulose; therefore, lower baseline ADL facilitates enhanced digesion, accelerating digestion rates compared to higher-fiber or highly lignified varieties. Although total mixed rations were formulated to be isonitrogenous (18.5% CP) to support targeted growth rates of up to 120 g/day, the intrinsic differences in forage fiber architecture among cultivars play a decisive role in modulating subsequent dry matter intake, rumen passage kinetics, and overall energy availability (Nascimento, 2009).

### 4.2 Voluntary feed intake and nutrient consumption dynamics

The supplementation of balanced concentrate rations significantly enhances roughage utilization, nutrient digestion, and metabolic efficiency in small ruminants (ARC, 1980). In the current study, total dry matter intake (TDMI) expressed as a percentage of body weight ranged between 2.42% and 2.57%, falling squarely within the standard physiological recommendations established for growing sheep (ARC, 1980). The marked increase in dry matter and nutrient consumption observed in lambs fed Treatment 3 (Avena sativa 5527A hay + concentrate) is driven by differences in forage cell-wall architecture and physical rumen fill constraints rather than variations in diet formulation. Although all total mixed rations were formulated to be isonitrogenous (18.5% CP) and isoenergetic (10.34 MJ/kg ME), genotype 5527A possessed a lower baseline fiber fraction (45.73% NDF, 30.76% ADF, and 4.31% ADL) compared to genotypes SRCPX80AB2806 and 5526A. Plant cell wall concentration, particularly NDF and acid detergent lignin, acts as the primary physical constraint regulating voluntary feed intake in high-forage ruminant diets (Van Soest, 1994; Allen, 2000). Lower cell wall lignification accelerates the rate of ruminal digestion and particle size reduction, and accelerating digesta passage kinetics (Keno et al., 2021; Ren et al., 2025). Consequently, reduced physical reticulorumen fill enables the animal to increase voluntary feed intake without encountering gut fill limitations (Haddad & Ata, 2009; Akhlaghi, 2024). The observed intake range of 158.96 to 186.42 g/d crude protein and 9.07 to 10.31 MJ/d metabolizable energy directly supported average daily gains of 70.00 to 114.95 g/d in Bonga lambs, matching standard production curves (ARC, 1980). These findings corroborate earlier observations by Wodebo et al. (2023), confirming that genetic variability in oat biomass yield, stem lignification, and cell wall chemistry significantly influences voluntary feed intake and livestock growth performance in Ethiopian highland environments.

### 4.3 Apparent digestibility of structural and soluble fractions

The variations observed in apparent nutrient and structural component digestibilities among the treatment groups stem directly from the intrinsic chemical composition and cell-wall architecture of the respective Avena sativa genotypes. Forage oats are widely recognized for supplying abundant water-soluble carbohydrates and digestible fiber fractions that influence total tract nutrient utilization (Hanoğlu Oral, 2024). Despite identical dietary crude protein (18.5%) and metabolizable energy (10.34 MJ/kg) levels across the total mixed rations, lambs fed Treatment 3 (Avena sativa 5527A) exhibited superior apparent digestibilities for dry matter (75.99%), organic matter (77.97%), NDF (61.99%), and ADF (54.01%).

This enhanced digestive efficiency is fundamentally governed by the structural recalcitrance of the plant cell walls. Because genotype 5527A possesses a lower proportion of acid detergent lignin (ADL = 4.31%) and neutral detergent fiber (NDF = 45.73%) compared to genotypes 5526A and SRCPX80AB2806, it provides a more accessible matrix for cellulolytic rumen colonization. Selecting oat genotype with lower fiber and better Crude protein containt (5527A) ubstantially improves the apparent digestibility of nutrients, driving superior performance in small ruminant production systems. Rowe et al.(2001) further report that lignin is genetically determined, allowing selection of low-lignin cultivars, with an expected digestible-energy advantage of approximately 1.5 MJ/kg dry matter and improved feed-conversion efficiency in animals fed low-lignin oats.

The clear nutritional advantage observed in sheep fed genotype 5527A (T3) is fundamentally driven by its lower baseline ADL (4.31%) and neutral detergent fiber content, which significantly reduces structural recalcitrance compared to more lignified cultivars (Wang et al., 2021). Consequently, the lower structural barrier in genotype 5527A accelerates overall fiber degradation kinetics and apparent nutrient digestibility without violating fundamental digestive principles.

### 4.4 *In vivo* growth kinetics

The marked improvements in final body weight, average daily gain (ADG), and feed conversion efficiency observed in lambs fed Treatment 3 underscore the profound impact of forage genotype selection on ruminant growth kinetics. Although all total mixed rations were formulated to be isonitrogenous (18.5% CP) and isoenergetic (10.34MJ/kg ME), lambs under the T3 regime achieved an ADG of 114.95 g/d compared to 87.99 g/d in T2 and 70.00 g/d in T1. This biological enhancement is directly linked to the superior cell-wall architecture and lower fiber and lignin content inherent to the Avena sativa ILRI_5527A variety used in T3. In similar controlled lamb study, reducing dietary NDF improved FCR from 5.39 to 4.33 without impairing ADG (Mirzaei-Alamouti et al., 2021). Low-fiber feed promotes higher intake, which drives increased crude protein consumption while minimizing energy expenditure per unit of gain. Consequently, treatment T3 achieved a significantly lower feed conversion ratio (8.69) and reduced protein and energy requirements per kilogram of gain. This is supported by Da Silva et al.(2016) roughage–concentrate study further found that higher roughage reduced energy and protein intake and compromised performance.T3 result also supported by other study that feed efficiency is also related to growth and energy expenditure in lambs (Zhang et al., 2021).

### 4.5 Correlation matrix and linear relationships among dietary intakes and performance variables

The exceptionally strong linear relationships (R^2^ = 1.000) observed between nutrient consumption vectors and live weight gains underscore the deterministic role of plane of nutrition in ruminant growth kinetics. When physical and chemical constraints within forage cell walls are minimized such as through the lower neutral detergent fiber (NDF) and acid detergent lignin (ADL) contents in Avena sativa genotype 5527A (T3) live weight gain scales directly and predictably with digestible protein and energy intake. These findings align with nutritional modeling principles emphasizing that nutrient flux dictates tissue deposition rates in growing sheep. While chopping forage to a uniform particle size standardizes physical intake across treatments, cultivar-specific chemical attributes remain the primary biological driver of metabolic efficiency and growth performance. This confirms earlier assertions that intrinsic cell-wall chemistry supersedes mechanical processing length in governing true nutritive value and animal productivity. Research evaluating diverse cereal and forage legume supplementation strategies highlights that improvements in nitrogen and metabolizable energy intake strongly correlate with average daily gain (da Cruz et al., 2021; Vahidi et al., 2021). Furthermore, investigations into cereal forage utilization confirm that reductions in structural fiber recalcitrance improve the precision of predictive growth models, allowing producers to forecast animal performance accurately based on dietary intake matrices (Akbar, N., 2018; Belanche et al., 2025).

### 4.6 Bio-Economic viability and financial sensitivity scenarios

The integration of biological efficiency and economic profitability is the ultimate criterion for recommending livestock technologies to smallholder farmers and commercial feedlot enterprises. The partial budget analysis clearly demonstrates that dietary inclusion of *Avena sativa* genotype 5527A combined with a balanced concentrate mix (T3) offers superior economic returns over genotypes 5526A (T1) and SRCPX80AB2806 (T2). These findings align with previous economic evaluations of small ruminant fattening systems in Ethiopia, which established that dietary interventions minimizing feed cost per kilogram of gain while maximizing market weight yield the highest financial advantage (Tulu et al., 2023).

In smallholder production systems, the treatment with the highest marginal rate of return and net benefit is considered the most economically viable option for adoption (Gebre et, a., 2020). In the present study, Treatment 3 yielded a net benefit of 33.75 USD per head and an MRR of 1.38, outperforming lower-quality forage diets where financial margins were nearly neutralized by high maintenance costs and poorer feed conversion ratios. Similar bio-economic advantages have been documented globally, where optimizing forage quality and nutrient synchronization in ruminant diets significantly reduces the cost of gain and elevates profitability under commercial market conditions (Yimenu, S.A. & Abebe, A., 2023). The superior economic performance of T3 stems directly from its biological efficiency; by minimizing structural cell-wall recalcitrance and enhancing nutrient intake, lambs achieved rapid average daily gains (114.95 g/d) with a reduced feed conversion ratio (8.69). This biological optimization lowered the overall feed cost per kilogram of gain to 0.13 USD, 0.10 USD, and 0.08 USD for T1, T2, and T3, respectively

To account for market volatility, financial sensitivity analyses were conducted across various adverse economic scenarios, including increases in total variable costs (10% and 20%) and decreases in gross income from lamb sales (10% and 20%), as well as combined negative shocks. The sensitivity analysis reveals that Treatment 3 maintains robust financial resilience, preserving a positive net benefit even under severe market fluctuations such as a 20% surge in input costs or a 20% drop in market prices. This stability underscores the risk-mitigating nature of utilizing high-yielding, superior-quality forage varieties like *Avena sativa* 5527A. Selecting high-yielding, highly digestible forage varieties like *Avena sativa* 5527A safeguards smallholder farmers against market price shocks, ensuring stable economic returns in intensive lamb production systems. These results corroborate economic evaluation frameworks established by CIMMYT (1988) and recent tropical livestock production studies (Hassen et al., 2022; Batista et al., 2018), confirming that biological optimizations in forage utilization translate directly into reliable, risk-resilient farm-gate profitability.

## 5 Conclusions

Based on the study, Treatment 3 (T3) consisting of *Avena sativa* 5527A hay with concentrate is the most effective dietary option for Bonga lamb. It significantly enhances total dry matter intake (997.98 g/d), apparent nutrient digestibility (75.99% DM), and average daily gain (114.95 *g*/d) compared to T1 and T2. Furthermore, T3 yields the highest net benefit (33.75 USD) and demonstrates excellent economic resilience in sensitivity analyses. Practically, adopting the T3 dietary regime offers livestock producers an optimal strategy to maximize small ruminant productivity and profitability. Integrating *Avena sativa* genotype 5527A into total mixed rations optimizes small ruminant productivity by synergistically enhancing nutrient composition, voluntary intake regulation, and total-tract digestibility, which directly translates into superior growth performance and economic resilience for smallholder production systems.

Several limitations should be noted, including the evaluation of restricted oat genotypes (5526A, SRCPX80AB2806, and 5527A) evaluated exclusively with Bonga lambs under a single environmental setting, which suggests caution before broadly generalizing these findings to other breeds and agro-ecological zones. Future research should investigate genotype 5527A performance with alternative livestock breeds, and assess long-term feeding impacts on carcass characteristics and meat quality.

## Declaration of generative AI and AI-assisted technoogies in the manuscript preparation process

During the preparation of this work, the authors used Gemni(OpenAI) to assist with the language editing and to suport the generation and refinement of data analysis code. After using this tool, the author(s) carefully reviewed the edited al content and take(s) full responsibility.

## Ethics declaration

This study was conducted in accordance with the ARRIVE (Animal Research: Reporting of In Vivo Experiments) guidelines. This study was approved by the Jimma University, College of Agriculture and Veterinary Medicine, Research and the Ethical Review Committee. (Approval No R: R/GS/922/2024)

## CRediT authorship contribution statement

**Kibreab Yosefe Wodebo:** Writing – review & editing, Writing – original draft, Visualization, Validation, Software, Resources, Project administration, Methodology, Investigation, Formal analysis, Data curation, Conceptualization. **Taye Tolemariam Ejeta:** Writing – review & editing, Visualization, Validation, Supervision, Investigation, Data curation, Conceptualization. **Weyessa Garedew Terefe:** Writing – review & editing, Visualization, Validation, Supervision, Investigation, Data curation, Conceptualization. **Muluken Zeleke Ekule:** Writing – review & editing, Visualization, Validation, Investigation, Data curation, Conceptualization. **Jane Ambuku Wamatu:** Writing – review & editing, Visualization, Investigation, Data curation, Conceptualization.

## Declaration of competing interest

The authors declare that they have no known competing financial interest or personal relationships that could have appeared to infuence the work reported in this paper

## Data Availability

Data will be made avaiabe by request
